# Supplementary material for: Irrigation of biomass plantations may globally increase water stress more than climate change
Source: Nat Commun. 2021 Mar 8;12:1512. doi: 10.1038/s41467-021-21640-3 (PMC7940422; doi:10.1038/s41467-021-21640-3)
Supplement: Supplementary file 1 — Supplementary Information [file 41467_2021_21640_MOESM1_ESM.pdf]

Supplementary Material: Irrigation of biomass plantations may globally increase water stress more than climate change

Fabian Stenzel[1,2,3,4,*], Peter Greve[2], Wolfgang Lucht[1,3,4], Sylvia Tramberend[2], Yoshihide Wada[2], and Dieter Gerten[1,3,4]

[1]*Potsdam Institute for Climate Impact Research (PIK), Member of the Leibniz Association, P.O. Box 60 12 03, D-14412 Potsdam, Germany*

[2]*International Institute for Applied Systems Analysis (IIASA), Schlossplatz 1, 2361 Laxenburg, Austria*

[3]*Humboldt-Universität zu Berlin, Department of Geography, Unter den Linden 6, D-10099 Berlin, Germany*

[4]*Integrative Research Institute on Transformations of Human-Environment Systems, Unter den Linden 6, D-10099 Berlin, Germany*

[*]*Corresponding author:* stenzel@pik-potsdam.de

This supplement contains additional information, which could not be included in the main manuscript. It also includes versions of Figures from the main manuscript for all GCMs. Additionally, this supplement contains Figures based on *max. yearly stress*, defined as the WSI of the mostly stressed month in a grid cell.

Supplementary Table 1 displays detailed globally aggregated data for all scenarios considered as the mean of all 4 GCMs.
Supplementary Table 2 shows GCMs specific data for the main scenarios (Today, CC, Baseline, BECCS, and BECCS+SWM).
Supplementary Figure 1 shows the GCM-specific maps for Figure 3a.
Supplementary Figure 2 shows the GCM-specific maps for Figure 3b.
Supplementary Figure 3 displays the grid-cell specific max. month WSI for HadGEM2-ES.
Supplementary Figure 4 displays the grid-cell specific max. month WSI as well as mean WSI for GFDL-ESM2M.
Supplementary Figure 5 displays the grid-cell specific max. month WSI as well as mean WSI for IPSL-CM5A-LR.
Supplementary Figure 6 displays the grid-cell specific max. month WSI as well as mean WSI for MIROC5.
Supplementary Figure 7 shows the differences in water stress in future scenario CC compared to today.
Supplementary Figure 8 shows the differences in water stress in future scenario BECCS compared to today.
Supplementary Figure 9 displays the precipitation differences between RCP2.6 and RCP6.0.
Supplementary Figure 10 shows the relative difference in area equipped for irrigation.
Supplementary Figure 11 displays the global crop harvest (excluding grassland, pastures, and bioenergy crops) per year for the 4 scenarios (CC, Baseline, BECCS, and BECCS+SWM) for

each GCM.

Supplementary Figure 12 displays that adding irrigation in the BECCS scenario has little effect on harvests compared to the Baseline (the curves are virtually identical). Limiting the irrigation water withdrawals in the BECCS+SWM scenario is approximately balanced by the increased on-field water use efficiency (total crop harvests are between 3% and 5% lower), which is much smaller than the variability induced by the different climate inputs (up to 18%).

Supplementary Figure 13 shows the comparison of water stress drivers between scenarios BECCS+SWM and CC.

Supplementary Figure 14 displays the gridcell specific area shares of food crops and pastures (green) overlain with those of bioenergy (red) for IPSL-CM5A-LR.

Supplementary Figure 15 displays the gridcell specific area shares of food crops and pastures (green) overlain with those of bioenergy (red) for GFDL-ESM2M.

Supplementary Figure 16 displays the gridcell specific area shares of food crops and pastures (green) overlain with those of bioenergy (red) for MIROC5.

Supplementary Figure 17 is a version of Figure 1 including the global values for max. yearly WSI.

Supplementary Figure 18 shows the month with the highest WSI for scenario BECCS.

Supplementary Figure 19 shows stress difference for max. yearly stress similar to Figure 3.

Supplementary Figure 20 shows main driver for max. yearly stress similar to Figure 4.

Supplementary Table 1: Globally aggregated biomass harvest, water withdrawals, and water stress indicators for all scenarios considered. Shown are total biomass harvest (GtC), total yearly freshwater withdrawals (domestic, industrial, irrigation of agricultural crops and bioenergy plantations; km^3/yr), as well as global area and population exposed to (very) high water stress, presented as inter-model mean derived under forcing from 4 GCMs. Water stress is analyzed for the month with maximum stress and the annual mean, respectively, for today (2006-2015), the RCP6.0 scenario representing climate change, and RCP2.6 scenarios assuming different irrigation and water management levels (2090-2099 average).

	**Cumulative bioenergy harvest [GtC]**	**Total freshwater withdrawals [km^3/yr]**	**Area under WSI>40% in at least one month [Mha]**	**Population under WSI>40% in at least one month [Mio]**	**Area under WSI>40% in the yearly mean [Mha]**	**Population under WSI>40% in the yearly mean [Mio]**
Today	0	3456	2250	3786	1023	2284
RCP6.0 0%	15	4707	2975	5978	1487	3997
RCP6.0 30% (*CC*)	15	4861	3096	6163	1580	4146
RCP2.6 0% (*Baseline*)	296	4571	2946	6043	1508	4109
RCP2.6 15%	309	4706	3488	6391	1784	4412
RCP2.6 30% (*BECCS*)	344	5231	3738	6486	1928	4583
RCP2.6 45%	374	5676	3890	6539	2018	4660
RCP2.6 60%	402	6063	4010	6571	2094	4702
RCP2.6 30% EFR	318	3705	2704	5811	1192	3655
RCP2.6 45% EFR	336	3935	2792	5904	1225	3708
RCP2.6 60% EFR	353	4134	2866	5952	1253	3753
RCP2.6 90% EFR	379	4459	2946	5998	1288	3788
RCP2.6 30% EFR WM	327	3675	2754	5872	1186	3605
RCP2.6 45% EFR WM (*BECCS+SWM*)	346	3913	2855	5949	1224	3661
RCP2.6 60% EFR WM	363	4120	2926	6006	1252	3703
RCP2.6 90% EFR WM	391	4457	3014	6060	1291	3759

Supplementary Table 2: GCM-specific results for the main scenarios from Table 1.

	Cumulative bioenergy harvest [GtC]	Total freshwater withdrawals [km^3/yr]	Area under WSI>40% in at least one month [Mha]	Population under WSI>40% in at least one month [Mio]	Area under WSI>40% in the yearly mean [Mha]	Population under WSI>40% in the yearly mean [Mio]
Today HadGEM2-ES	0	3491	2172	3751	982	2229
Today MIROC5	0	3519	2318	3857	1065	2322
Today GFDL-ESM2M	0	3417	2273	3748	1026	2273
Today IPSL-CM5A-LR	0	3396	2237	3786	1020	2312
CC HadGEM2-ES	14	5047	3151	6276	1579	4242
CC MIROC5	18	4845	3024	6047	1520	4025
CC GFDL-ESM2M	13	4819	3158	6258	1607	4161
CC IPSL-CM5A-LR	14	4733	3051	6072	1613	4156
Baseline HadGEM2-ES	310	4618	2985	6072	1537	4203
Baseline MIROC5	313	4637	2955	5953	1523	4126
Baseline GFDL-ESM2M	273	4476	2909	6084	1481	4048
Baseline IPSL-CM5A-LR	290	4554	2936	6064	1490	4061
BECCS HadGEM2-ES	355	5238	3750	6493	1938	4647
BECCS MIROC5	359	5318	3803	6470	1970	4705
BECCS GFDL-ESM2M	318	5105	3653	6512	1901	4520
BECCS IPSL-CM5A-LR	342	5265	3747	6470	1903	4460
BECCS+SWM HadGEM2-ES	358	3984	2884	5987	1224	3787
BECCS+SWM MIROC5	364	4146	3010	6096	1327	3851
BECCS+SWM GFDL-ESM2M	318	3695	2734	5849	1167	3472
BECCS+SWM IPSL-CM5A-LR	343	3827	2792	5867	1178	3534

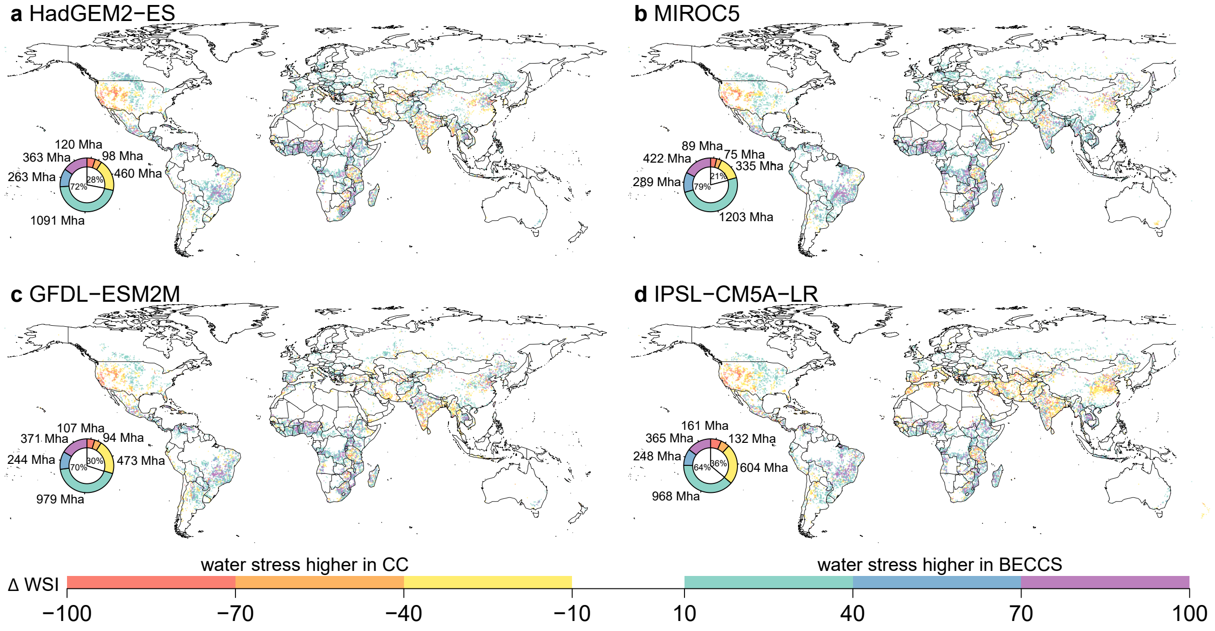

Supplementary Figure 1: As Figure 3a, but for all four GCMs.

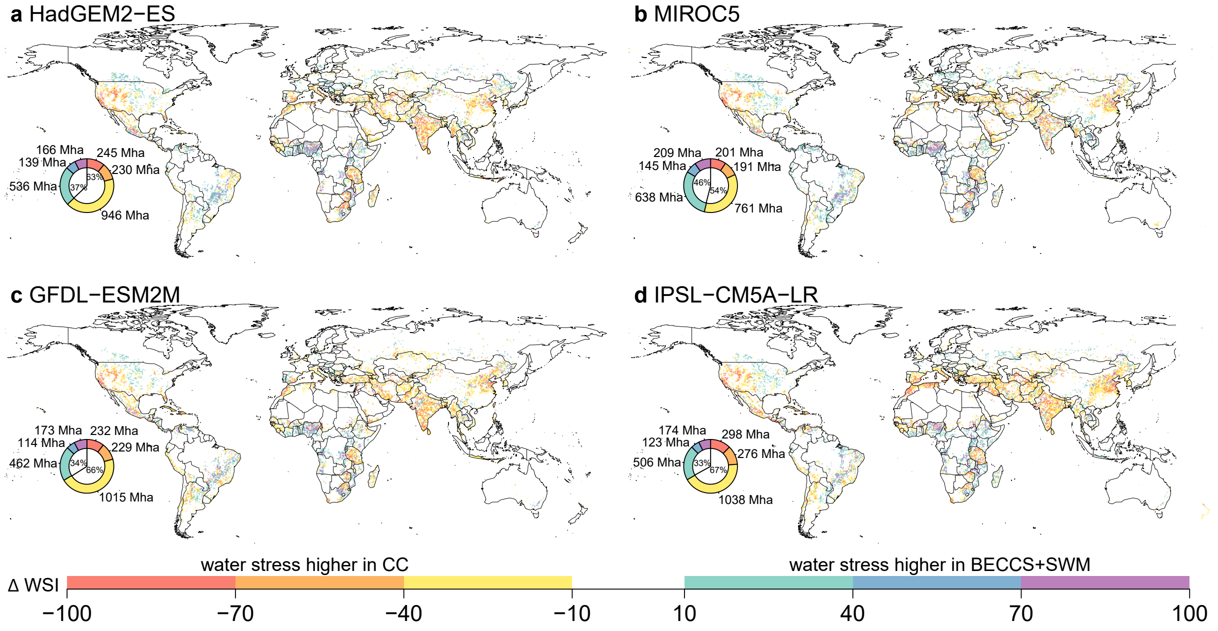

Supplementary Figure 2: As Figure 3b, but for all four GCMs.

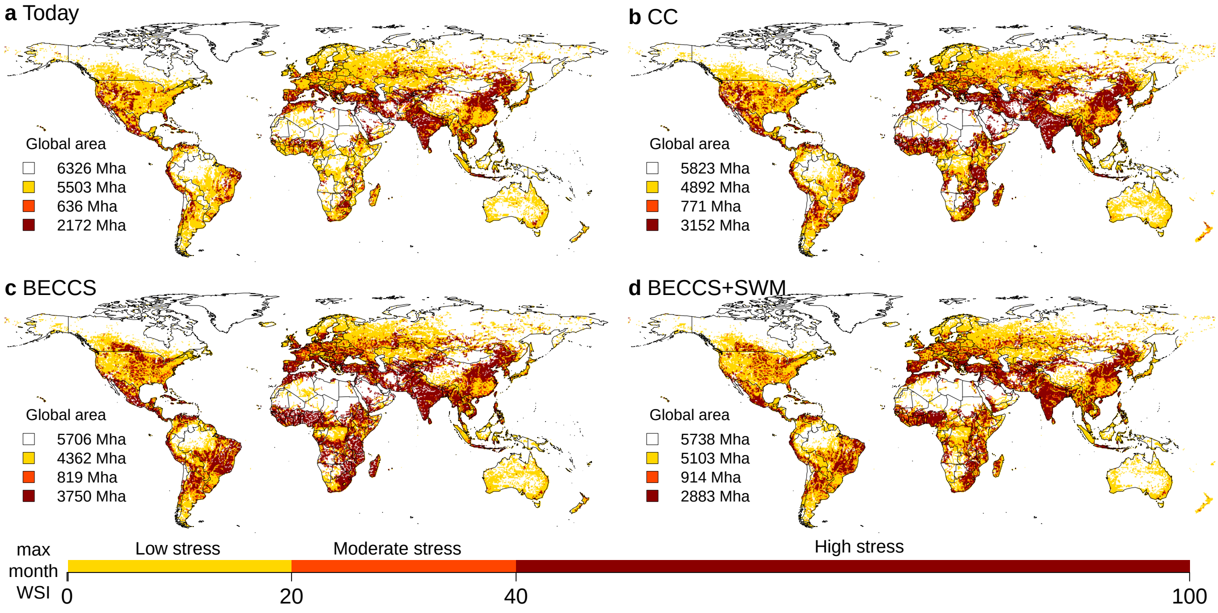

Supplementary Figure 3: As Figure 2, but for the water stress in the maximum month (HadGEM2-ES).

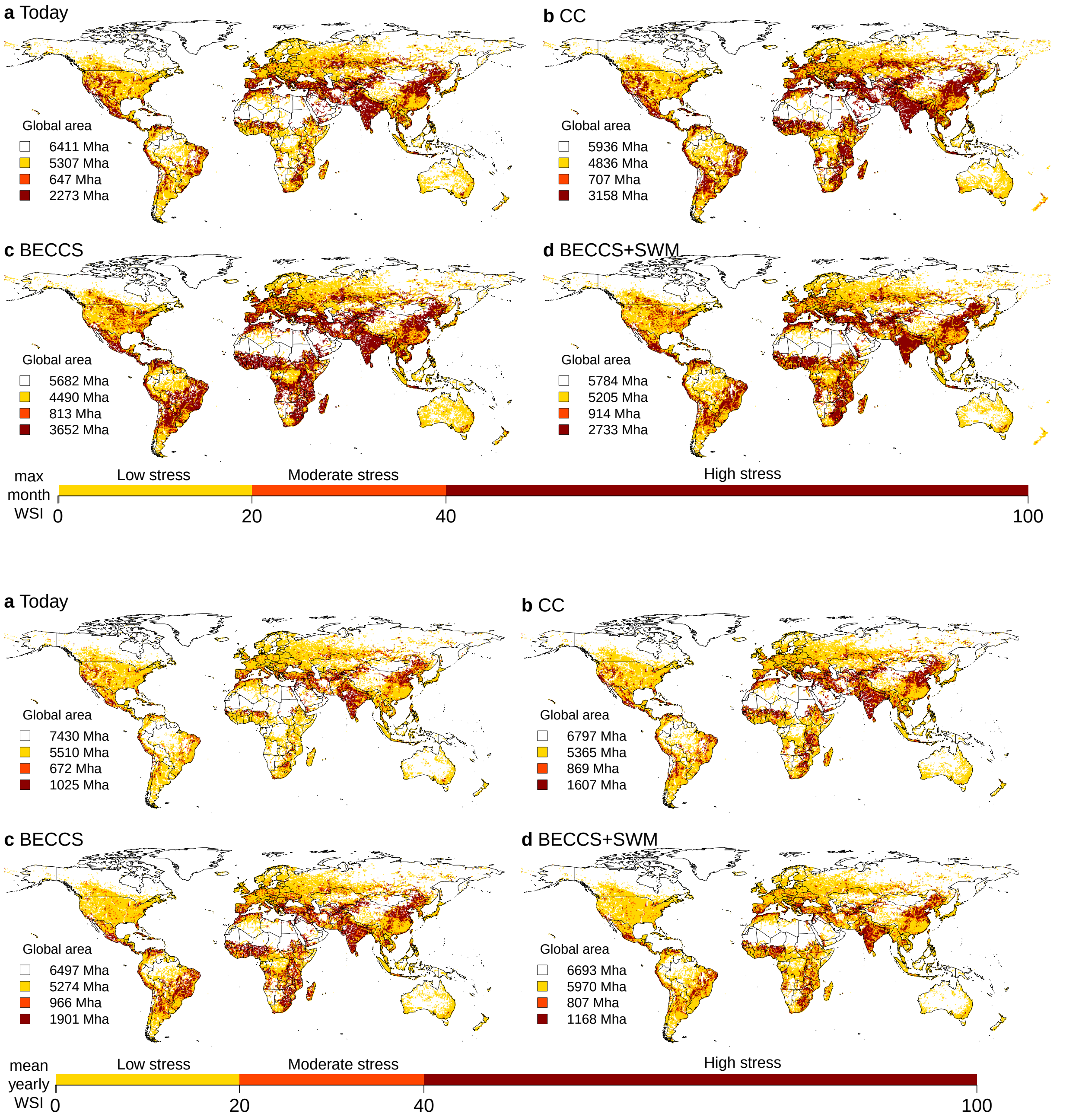

Supplementary Figure 4: As Figure 2 and Figure 3, but for GFDL-ESM2M. Top: maximum month water stress. Bottom: mean yearly water stress.

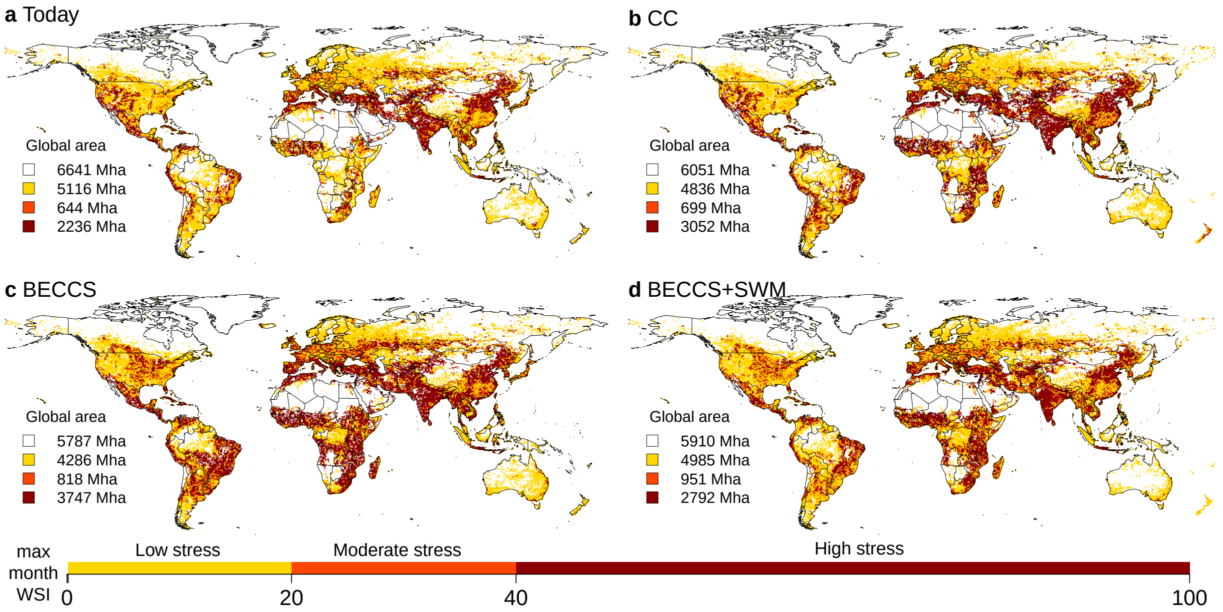

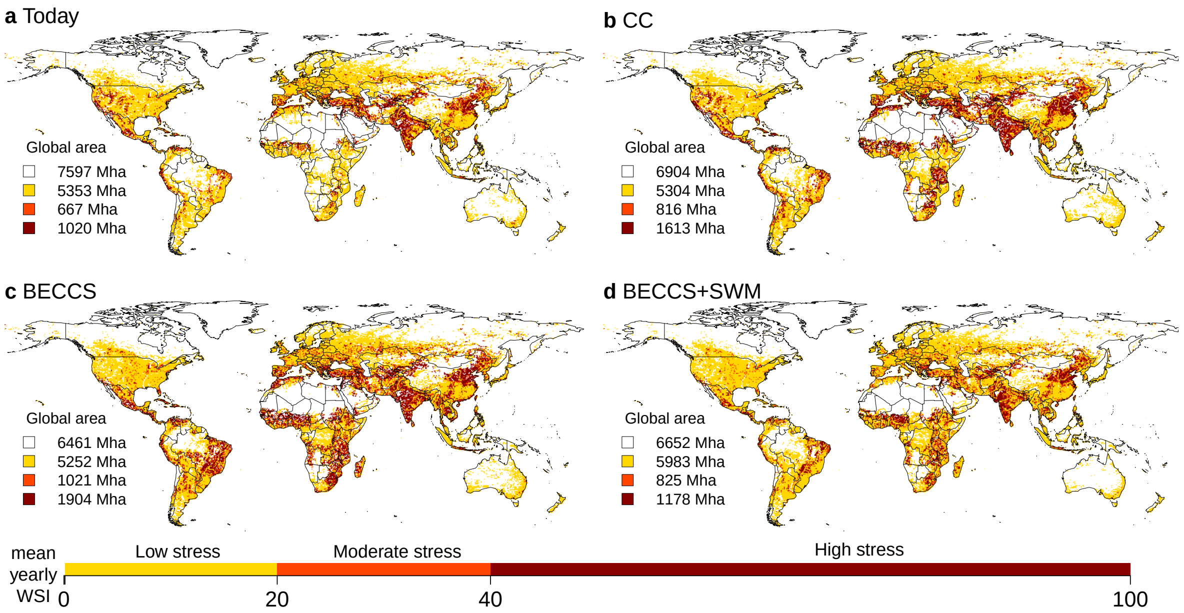

Supplementary Figure 5: As Figure 2 and Figure 3, but for IPSL-CM5A-LR. Top: maximum month water stress. Bottom: mean yearly water stress.

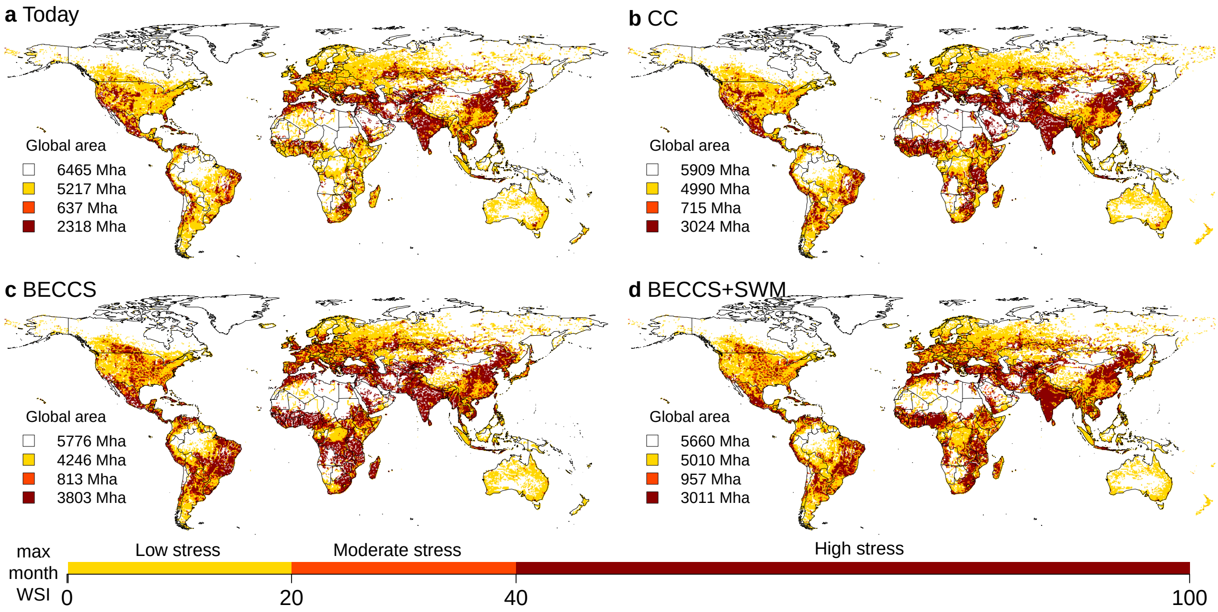

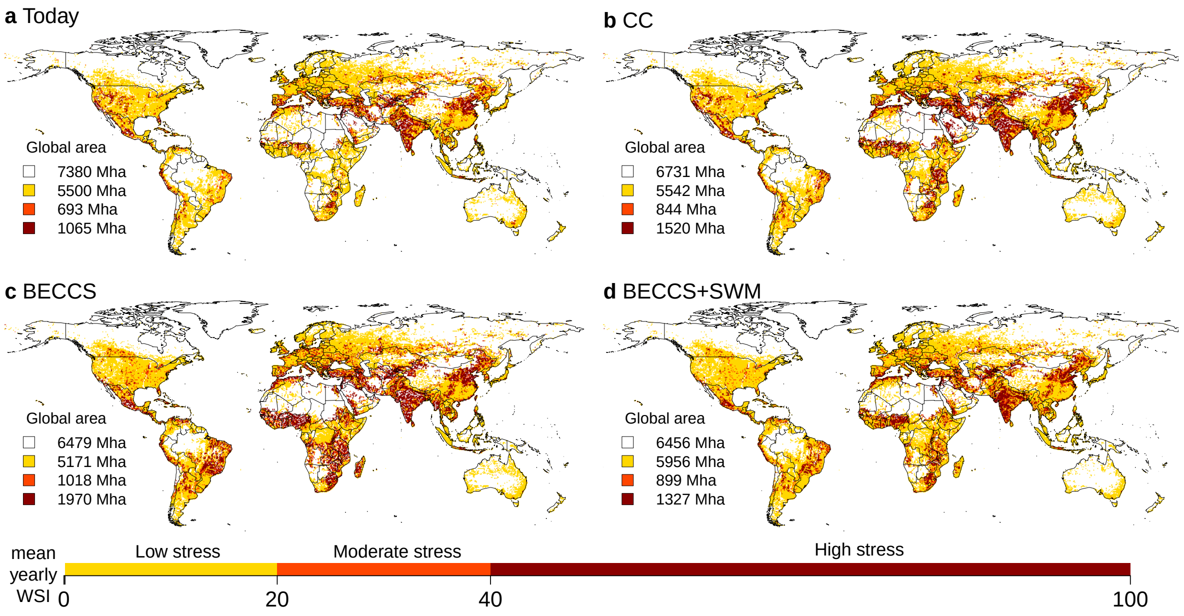

Supplementary Figure 6: As Figure 2 and Figure 3, but for MIROC5. Top: maximum month water stress. Bottom: mean yearly water stress.

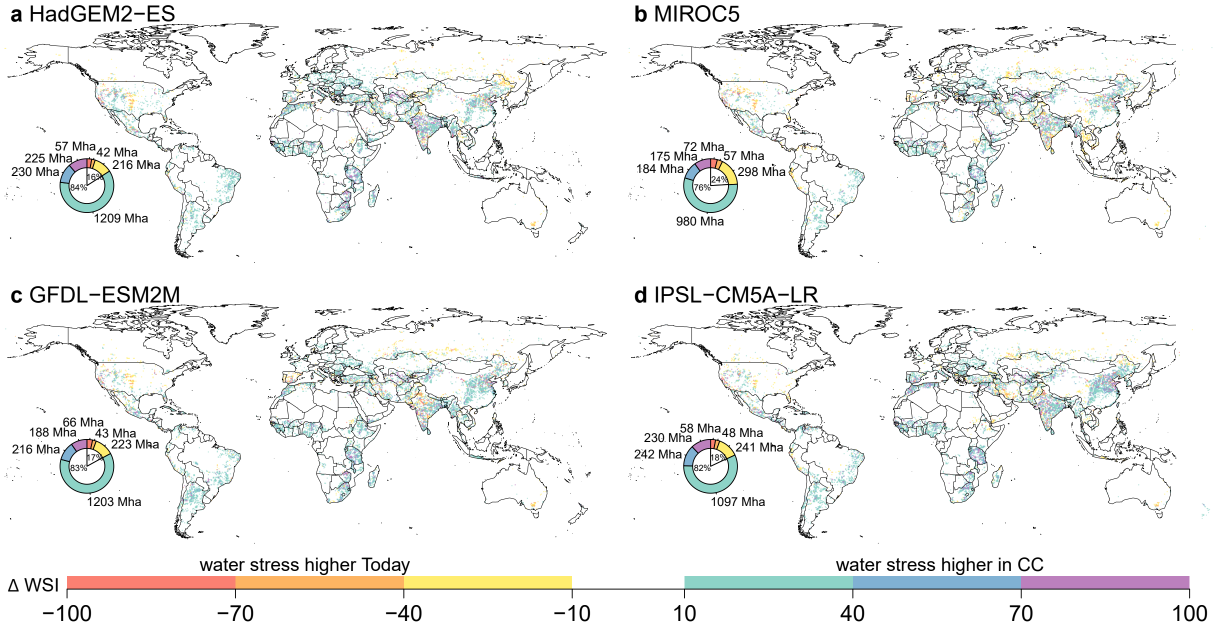

Supplementary Figure 7: Differences in water stress in future scenario CC compared to today. Shown are differences in WSI values for all four GCMs (2090-2099 average) in comparison to present conditions (2006-2015 average). Pie diagrams show the total area of each color-class.

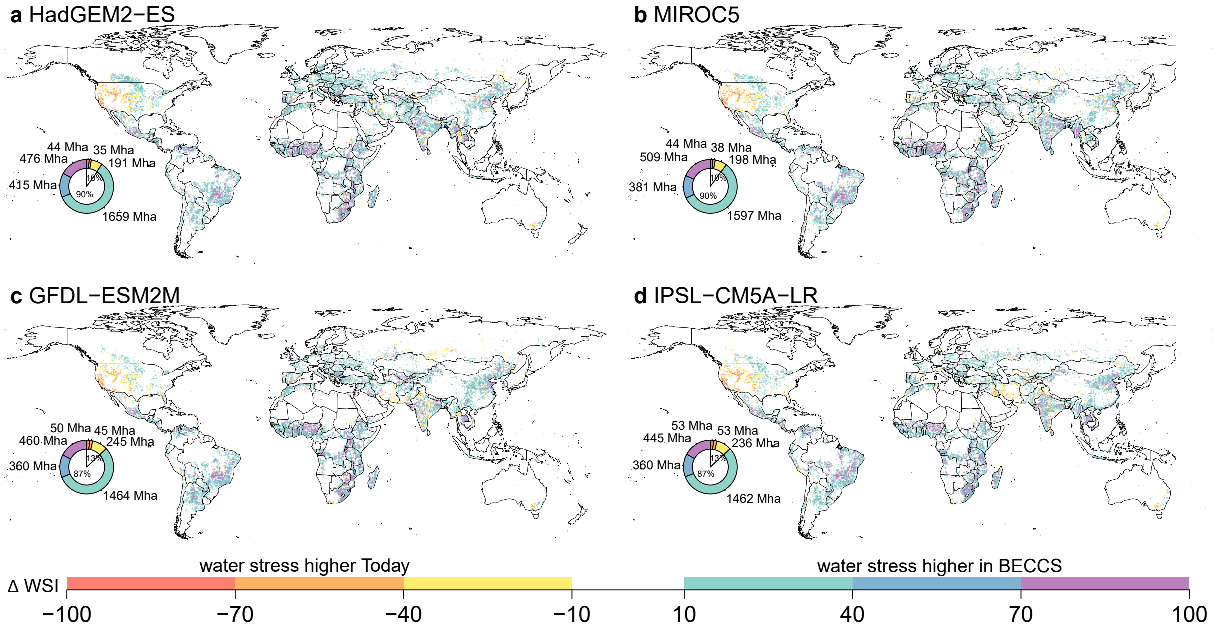

Supplementary Figure 8: Differences in water stress in future scenario BECCS compared to today. As Figure 7, but comparing WSI in scenario BECCS with today.

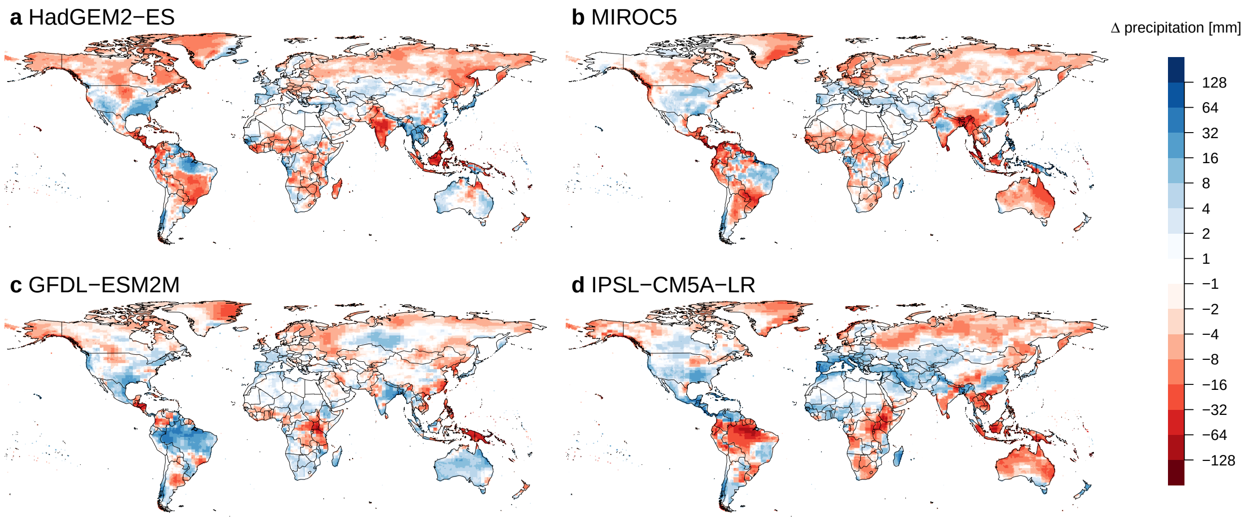

Supplementary Figure 9: Precipitation differences between RCP2.6 and RCP6.0 for the 4 GCMs considered. Shown are the absolute differences of the mean yearly precipitation from 2090–2099 provided by the ISIMIP2b project. Blue locations show higher precipitation in RCP2.6 and red locations in RCP6.0.

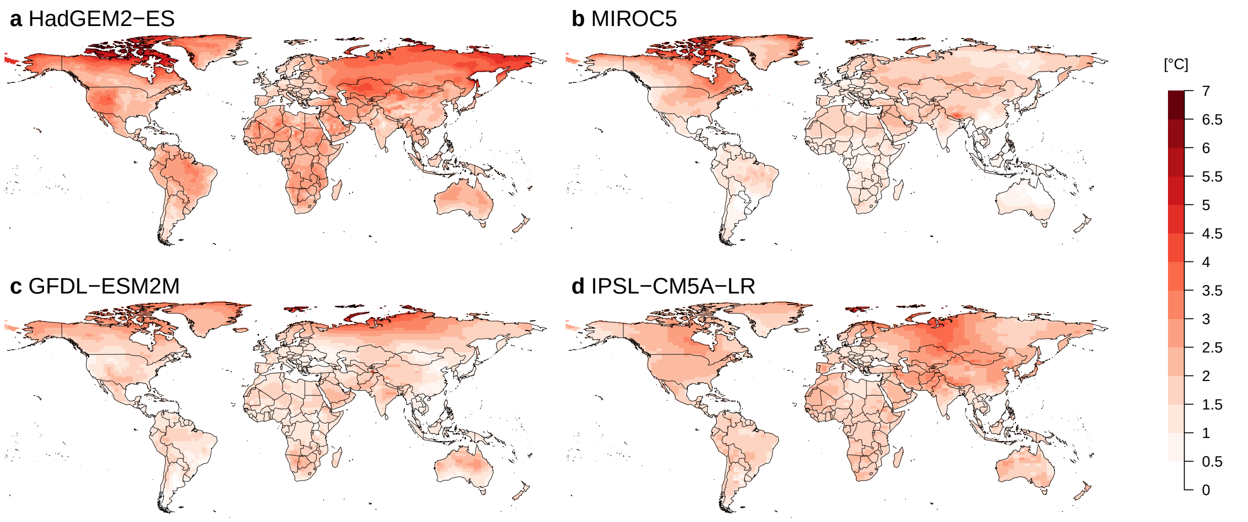

Supplementary Figure 10: Mean yearly Temperature difference (2090-2099) between RCP6.0–RCP2.6 for all four GCMs. Data from ISIMIP2b project.

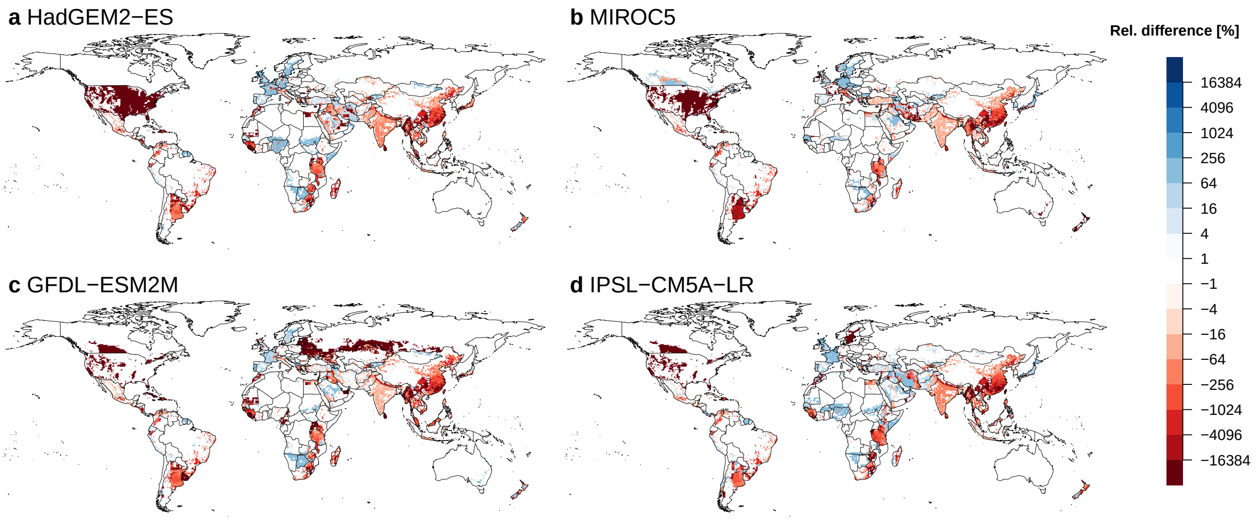

Supplementary Figure 11: Relative difference in area equipped for irrigation: (RCP2.6–RCP6.0)/RCP2.6x100. Difference of grid-cell specific sum of area equipped for irrigation (crops and pastures, excluding bioenergy crops) in 2095 (ISMIP2b) between RCP2.6 and RCP6.0 for all GCMs. Blue represents locations with larger irrigated areas in RCP2.6, while red locations show larger irrigated areas in RCP6.0.

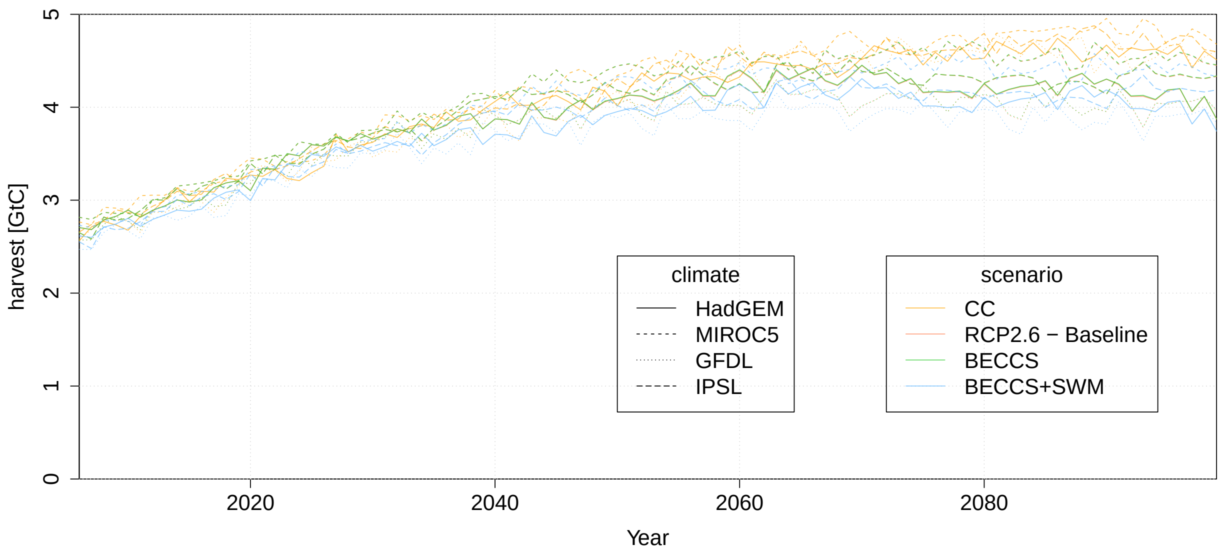

Supplementary Figure 12: Global crop harvest (excluding grassland, pastures, and bioenergy crops) per year for scenarios *CC*, *RCP2.6 - Baseline*, *BECCS*, and *BECCS+SWM* for the GCMs HadGEM2-ES, MIROC5, GFDL-ESM2M and IPSL-CM5A-LR. The harvest is calculated as the cft-specific LPJmL yield multiplied with the assumed productivity increases from MAgPIE. Values for *RCP2.6 - Baseline* and *BECCS* are virtually identical.

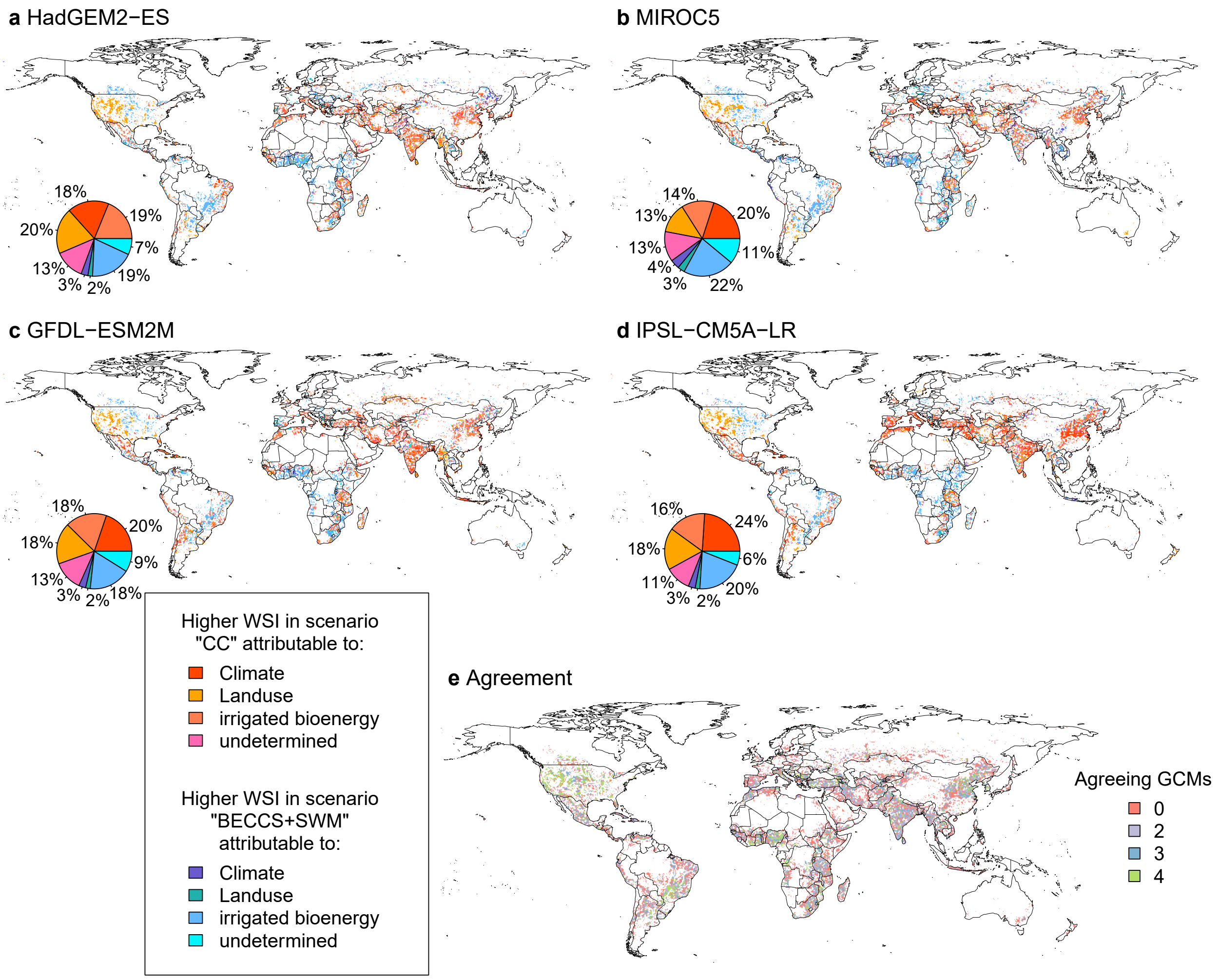

Supplementary Figure 13: As Figure 4, but showing the comparison of water stress between scenarios *BECCS+SWM* and *CC*.

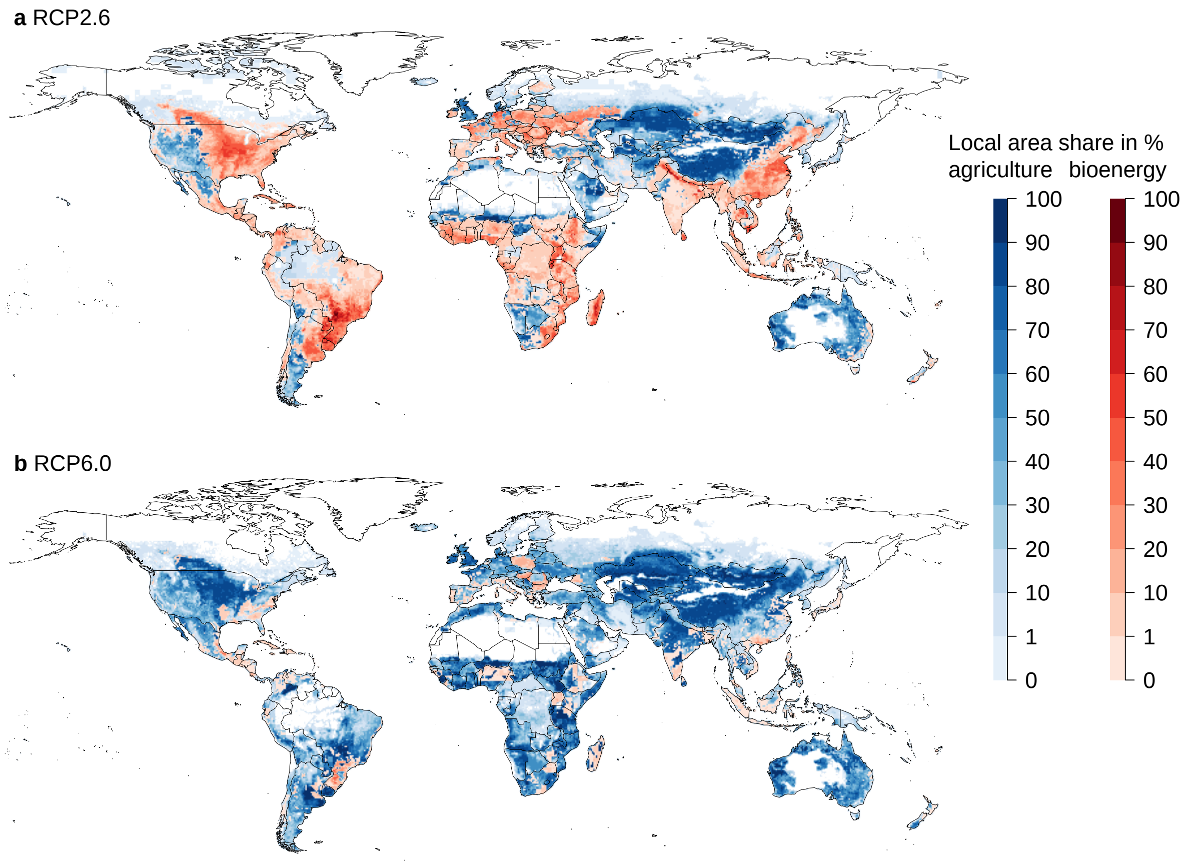

Supplementary Figure 14: Grid cell area shares of food crops and pastures (green) overlain with those of bioenergy (red) for 2090-2099 in the associated land use scenarios for RCP2.6 and RCP6.0 (623/32 Mha) in ISIMIP2b for the GCM IPSL-CM5A-LR.

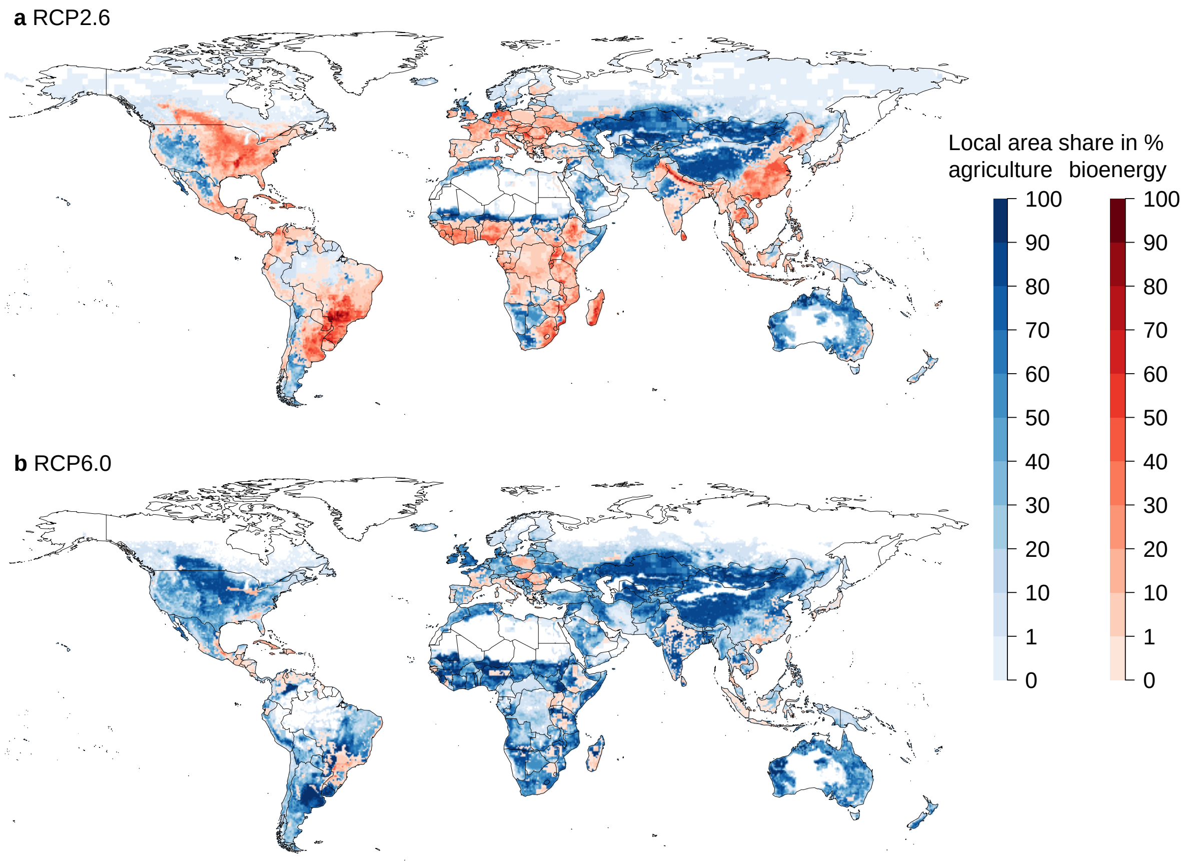

Supplementary Figure 15: Grid cell area shares of food crops and pastures (green) overlain with those of bioenergy (red) for 2090-2099 in the associated land use scenarios for RCP2.6 and RCP6.0 (596/28 Mha) in ISIMIP2b for the GCM GFDL-ESM2M.

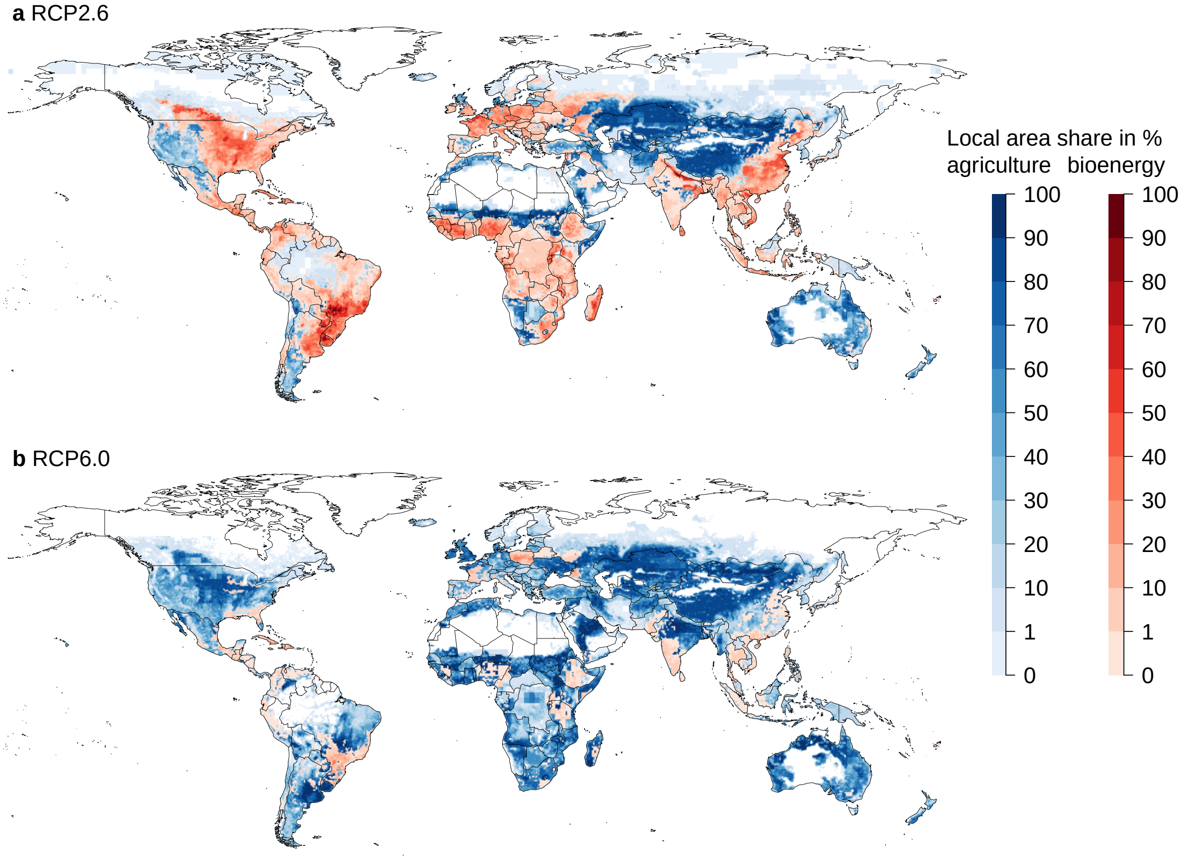

Supplementary Figure 16: Grid cell area shares of food crops and pastures (green) overlain with those of bioenergy (red) for 2090-2099 in the associated land use scenarios for RCP2.6 and RCP6.0 (592/32 Mha) in ISIMIP2b for the GCM MIROC5.

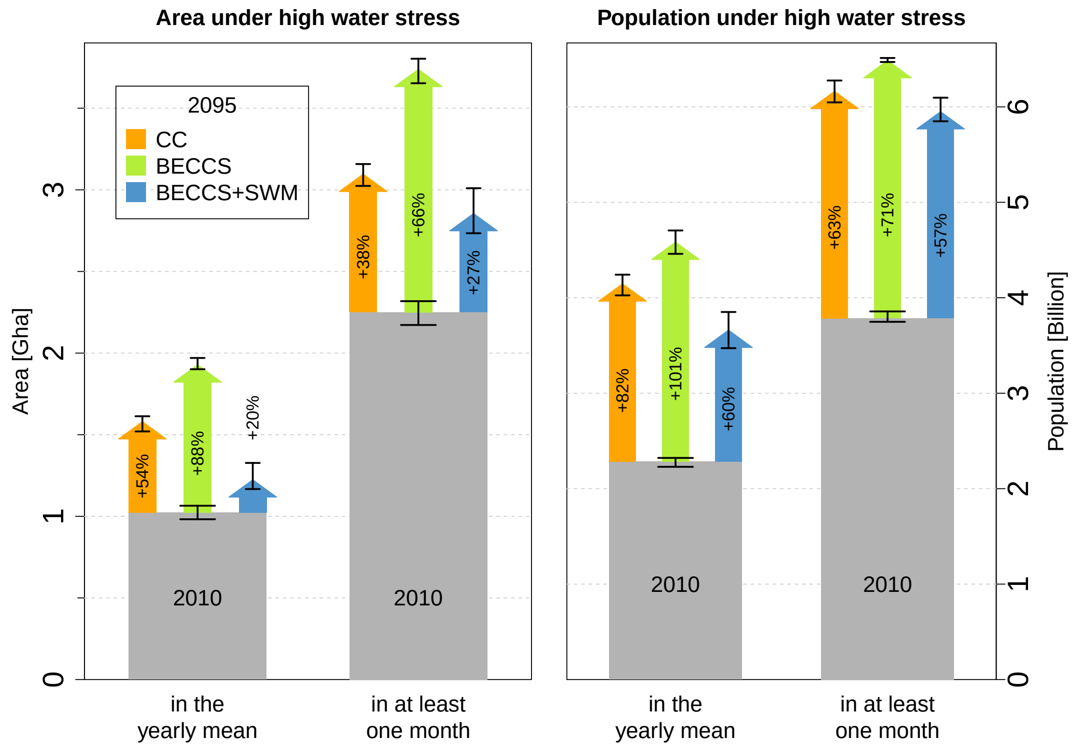

Supplementary Figure 17: Simulated increase of area and population exposed to high water stress from 2010 (2006-2015) to 2095 (2090-2099) in the different scenarios. The numbers represent global sums of grid cell-level area and population, respectively, where annual mean WSI>40% (left bars of each panel), or where WSI>40% in at least one month per year – max. month (right bars). Shown are the mean change and the ranges resulting from the differences in climate simulations based on the four GCMs. Grey bars represent the current (2006-2015 average) levels.

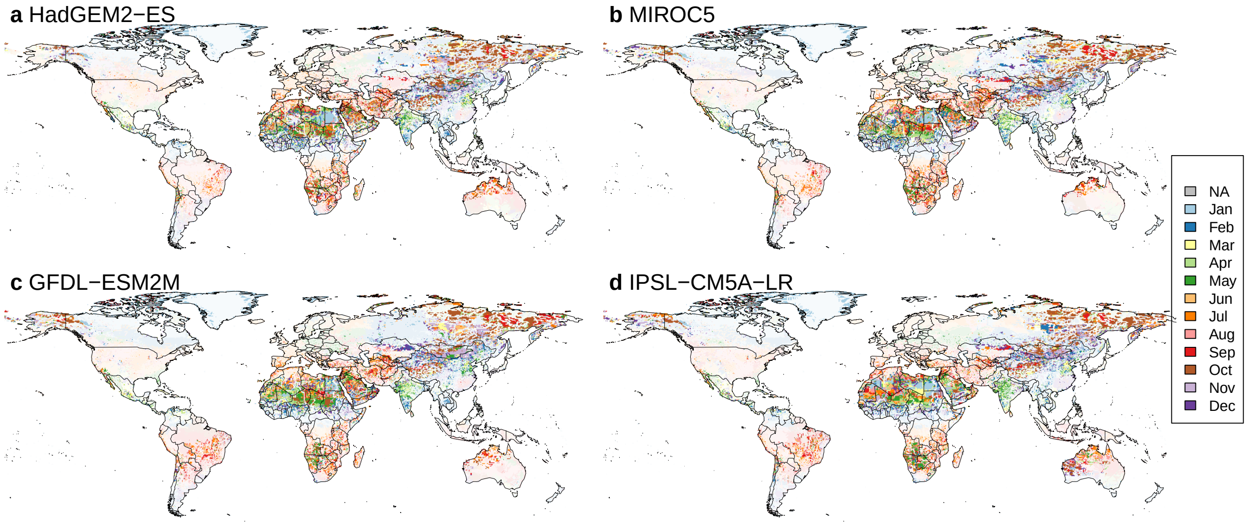

Supplementary Figure 18: Month with max. water stress for 2090-2099 in scenario *BECCS*. Five transparency levels are based on the local grid cell WSI (0-100%), whereby colors have no transparency (0) for WSI 70-100% and high transparency (0.9) for 0-20%. Intermediate steps are WSI 20-40% (transparency 0.7) and 40-70% (transparency 0.4).

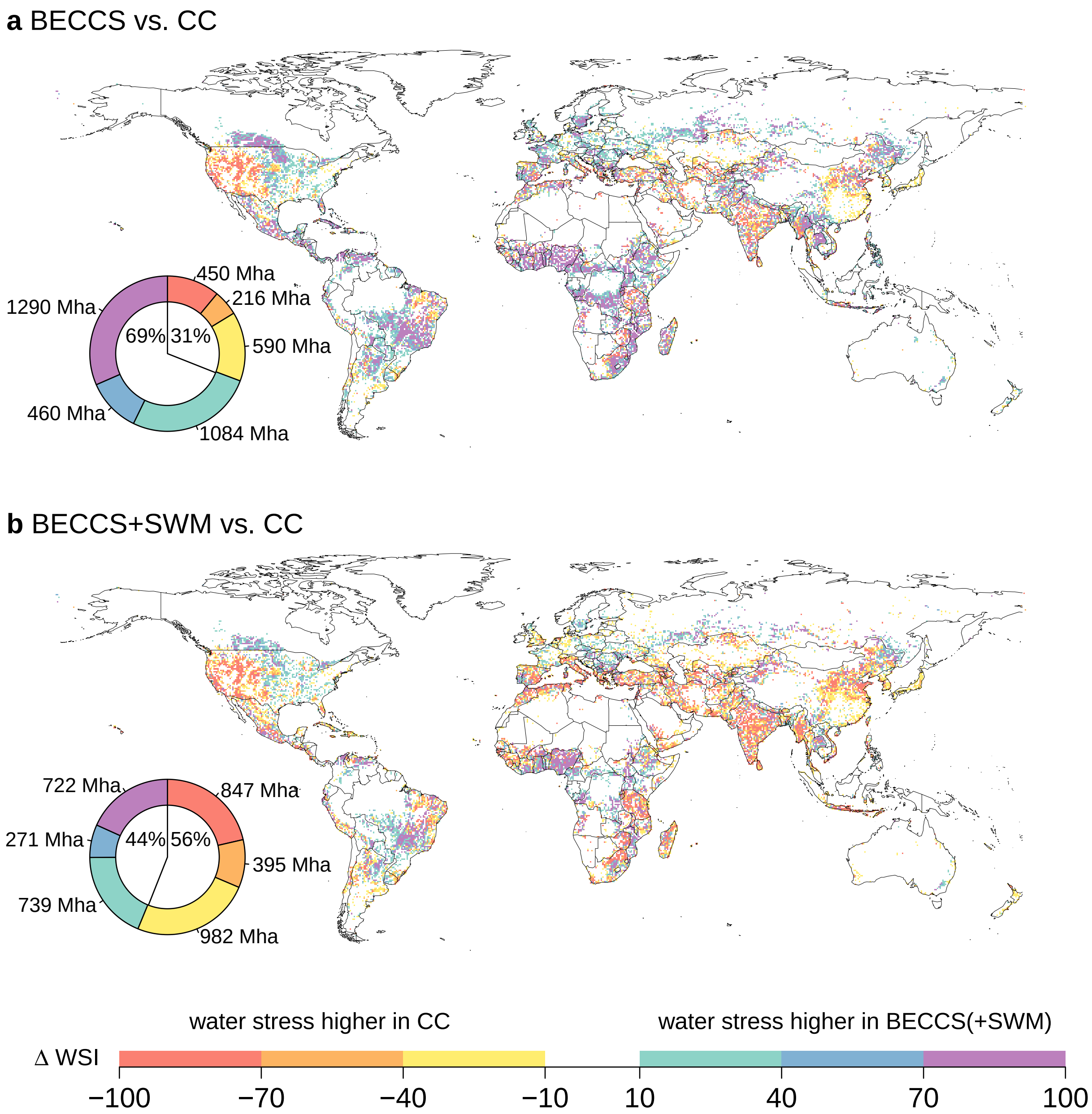

Supplementary Figure 19: Differences in peak water stress between scenarios BECCS(+SWM) and CC. As Figure 3, but for max. yearly water stress. Shown are differences in mean yearly WSI values (percentage points) among the different scenarios (here, under HadGEM2 climate forcing, 2090-2099 average). Pie diagrams show the total global area showing a certain (respectively colored) difference.

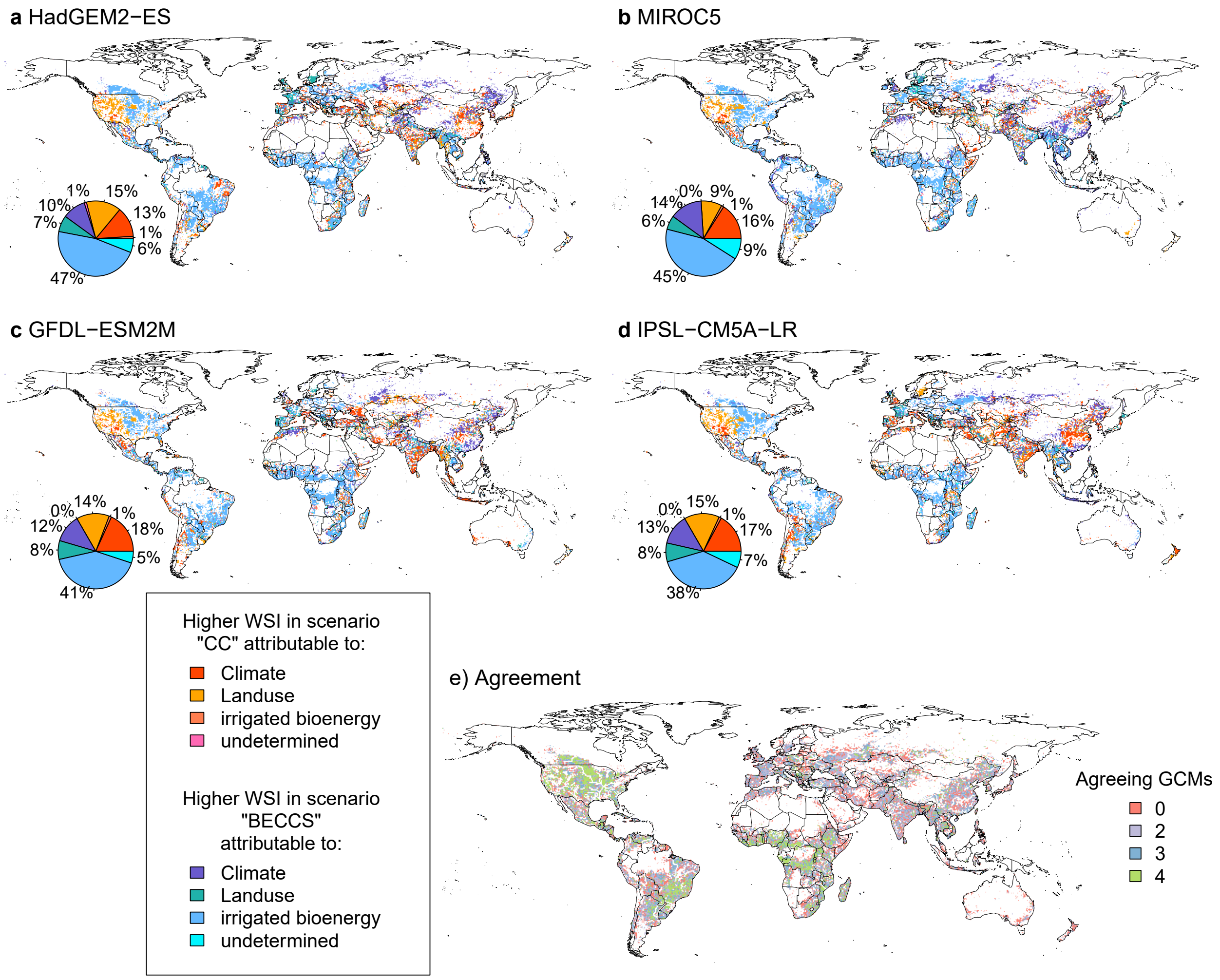

Supplementary Figure 20: Attribution of main driver explaining differences in peak water stress between the scenarios *BECCS* and *CC*. As Figure 4, but for max. yearly water stress. (a-d) Higher water stress in *BECCS* is indicated by blueish colors, the opposite in reddish colors. Drivers are attributed by factorial simulation experiments keeping either land use, climate or irrigation on biomass plantations constant (see Supplementary Online Methods – Attribution of drivers for water stress differences). The global area shares of each category are displayed to the bottom-left of each map. (e) Number of GCMs that agree on the attributed driver in a grid cell.
